# The impact of natural transformation on adaptation in spatially structured bacterial populations

**DOI:** 10.1186/1471-2148-14-141

**Published:** 2014-06-20

**Authors:** Danesh Moradigaravand, Jan Engelstädter

**Affiliations:** 1 Institute of Biogeochemistry and Pollutant Dynamics, ETH Zürich, 8092 Zürich, Switzerland; 2 School of Biological Sciences, The University of Queensland, Brisbane, QLD 4072, Australia

**Keywords:** Bacterial adaptation, Bacterial transformation, Biofilm, Spatially structured populations

## Abstract

**Background:**

Recent studies have demonstrated that natural transformation and the formation of highly structured populations in bacteria are interconnected. In spite of growing evidence about this connection, little is known about the dynamics of natural transformation in spatially structured bacterial populations.

**Results:**

In this work, we model the interdependency between the dynamics of the bacterial gene pool and those of environmental DNA in space to dissect the effect of transformation on adaptation. Our model reveals that even with only a single locus under consideration, transformation with a free DNA fragment pool results in complex adaptation dynamics that do not emerge in previous models focusing only on the gene shuffling effect of transformation at multiple loci. We demonstrate how spatial restriction on population growth and DNA diffusion in the environment affect the impact of transformation on adaptation. We found that in structured bacterial populations intermediate DNA diffusion rates predominantly cause transformation to impede adaptation by spreading deleterious alleles in the population.

**Conclusion:**

Overall, our model highlights distinctive evolutionary consequences of bacterial transformation in spatially restricted compared to planktonic bacterial populations.

## Background

Bacterial populations often live in highly structured and dense populations, known as biofilms, in nature [1]. Biofilms are stable assemblages of bacterial cells that stick to a surface and are embedded in a self-secreted matrix [1-3]. The proximity between cells is advantageous to the bacteria as it allows them to respond to environmental stresses more effectively, coordinate their behavior better and gain access to nutrients and public goods. The proximity can, however, be disadvantageous as it intensifies the competition for nutrients and other resources between the bacteria [2,4].

In natural habitats, formation of structured bacterial communities can occur concurrently with colonization of new environments and bacterial adaptation. In the course of colonization, bacteria may be under natural selection to adapt to their new environments. One way in which the necessary genetic variation for natural selection to act upon can be generated is natural transformation, the active uptake of naked DNA fragments from the environment and their subsequent integration into the genome. Natural transformation has been reported in many species of bacteria, including some important pathogens like *Streptococcus pneumoniae* and *Helicobacter pylori*[5].

There is growing evidence that biofilm formation and natural transformation are interconnected processes reviewed in [6]. The physical proximity of bacteria in space seems to facilitate horizontal gene transfer between them, as shown in *S. pneumoniae*[7] and *H. pylori*[8]. Further, protein factors that induce natural transformation were found to also trigger biofilm formation, leading to an increased rate of transformation in the biofilm compared to the planktonic state in naturally transforming bacteria [9-11]. Finally, extracellular DNA is known to be abundant in biofilms and also to be involved in biofilm formation [12,13].

Transformation has the potential to facilitate adaptation, owing to the creation of co-adapted combinations of genes [14-18] but also the acquisition of beneficial genes. However, some hypotheses for the evolution of natural transformation emphasize physiological advantages to transformation and regard any genetic effect as the by-product of these physiological benefits [19,20]. Natural transformation can also become costly depending on the composition of DNA fragments in the environment [15,21]: while acquiring new genes can enhance the fitness of the adapting bacteria, transformation with defective DNA that originate from bacteria that are selected against by natural selection can impede adaptation. The interplay between costs and benefits of transformation and bacterial adaptation has been investigated in some previous studies [15,21,22]. These studies, however, assumed that bacteria live in a planktonic state, and therefore, neglected the effect of spatial structure of bacterial populations. Since spatial restriction on population growth limits the availability of free DNA to bacteria, we expect the impact of transformation on population dynamics to be different in spatially restricted compared to the planktonic populations.

To study the connection between transformation with external DNA and structured population growth in bacteria, we developed a mathematical model for adaptation of a bacterial population during growth across space. The bacterial population was assumed to undergo transformation with DNA fragments released by dead bacteria. This eco-evolutionary model incorporates several ecological and population genetic factors, such as bacterial migration and DNA diffusion, that govern the rate of both population expansion and adaptation in space. We demonstrate how the impact of transformation on adaptation varies in different parts of a growing population, depending on DNA diffusion, bacterial migration and the source of free DNA in the environment.

## Results

### The dynamics of bacterial growth and DNA diffusion

**Figure 1:**
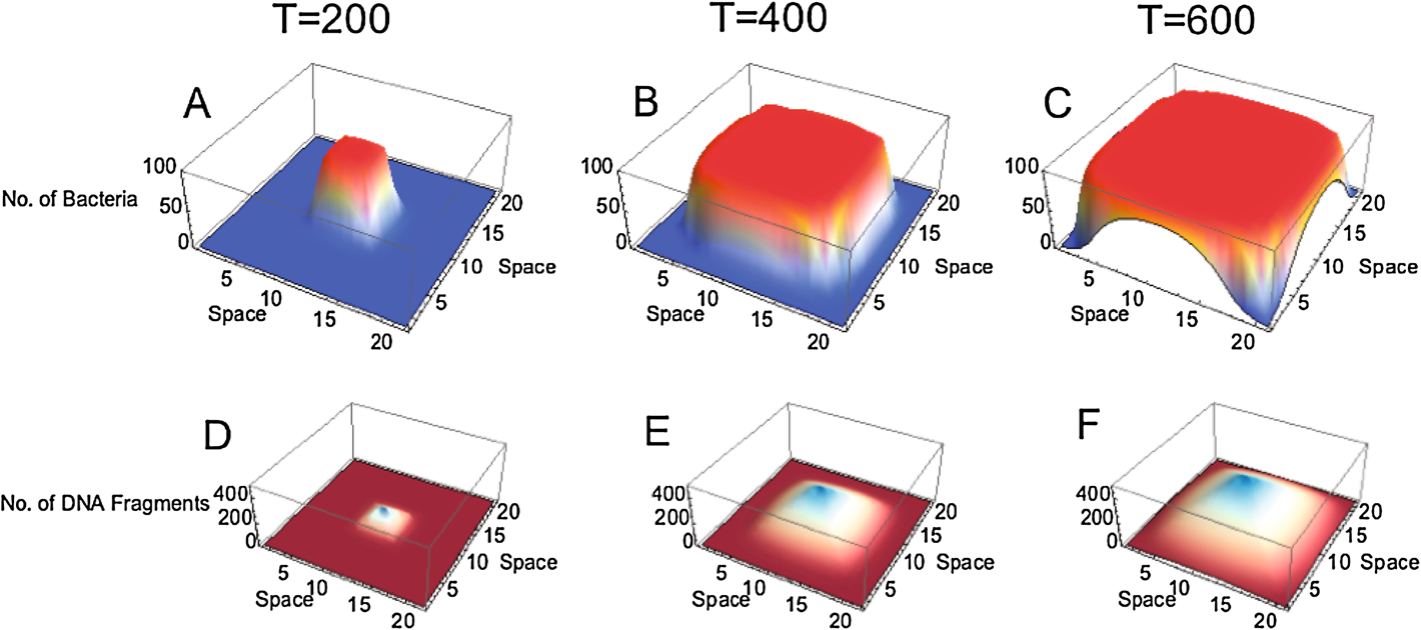
**Spatial distribution of a growing bacterial population and DNA fragment pool in a 21X21 grid space over time at three time points: A,D) *T*** **= 200, B,E)*T*** **= 400and C,F)*T*** **= 500.** Simulations start with a bacterium of genotype 0 located at the center of the space. Interpolation of order 1 was used to generate continuous plots from discrete data. Parameters take values *r* = 0.1, *K* = 100, *m* = 10^- 2^, *v* = 10^- 4^, *s* = 0, *u* = 0, *μ* = 0, *g* = 0, *c* = 0.

We first investigated the dynamics of a bacterial population expanding in space in the absence of adaptation. Figure 1 shows how a single bacterium gives rise to a bacterial colony across space over time. The population expands symmetrically because bacteria invade adjacent grid cells at the same rate (Figure 1A,B and C). Population growth stops when all grid cells are filled (Figure 1C). Depending on the migration and growth rates, bacteria may migrate to adjacent cells before the carrying capacity of a focal grid cell is reached. In this case, a less compact population structure is formed over time (not shown). Not surprisingly, high levels of bacterial migration lead to dynamics resembling those in a well-mixed planktonic population.Upon death, bacteria release DNA fragments into the environment. In the absence of DNA diffusion, the free DNA remains at the release location and accumulates with time. Since the free DNA has more time to amass in the initial grid cell, the DNA concentration is higher there (Figure 1D,E and F). In our simulations, there is always some levels of DNA diffusion and/or DNA degradation so that free DNA does not indefinitely accumulate in the environment.

### Adaptation with constant selection

**Figure 2:**
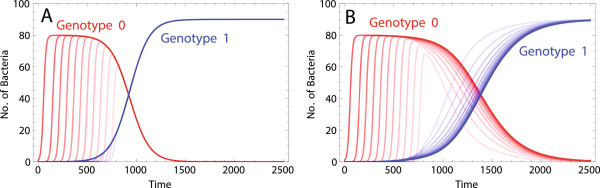
**The number of bacteria of genotype 0 and 1 in different grid cells of the equator section of a 21 × 21 space at one time point during adaptation in the absence (A) and presence (B) of transformation.** Simulations start with a bacterium of genotype 0 located at the center of the space. Red and blue curves correspond to the bacterial numbers of genotype 0 and 1, respectively, and curves corresponding to more centrally located grid cells are shown with stronger colors. Parameters take values *r* = 0.1, *K* = 100, *m* = 10^- 2^, *v* = 10^- 4^, *s* = 0.01, (*A*) *u* = 0 and (*B*) *u* = 10^- 5^, *μ* = 10^- 6^, *g* = 0.1, *c* = 0.

We next considered a growing bacterial population that adapts to a new environment under a constant selection regime where allele 1 has a constant selective advantage *s* over allele 0. The population starts from a bacterium of genotype 0, which gives rise to an expanding population. As the population grows, mutation creates bacteria of genotype 1, which in the absence of transformation go quickly to fixation (Figure 2A). Transformation delays the fixation of the beneficial allele in the population and impedes adaptation (Figure 2B). This occurs because the DNA pool during adaptation is overrepresented by the free DNA of type 0 released from the less fit bacteria of genotype 0. This so-called ‘bad genes effect’ of transformation, also described in prior studies [15,21], is more pronounced in central layers of the population. This leads to the formation of a frequency gradient of bacteria of genotype 1 across different layers of the growing population (Figure 2B). In this gradient, the frequency of bacteria of genotype 1 is lower in the central layers of the population in the short term, because of the higher concentration of the free DNA of type 0.

**Figure 3:**
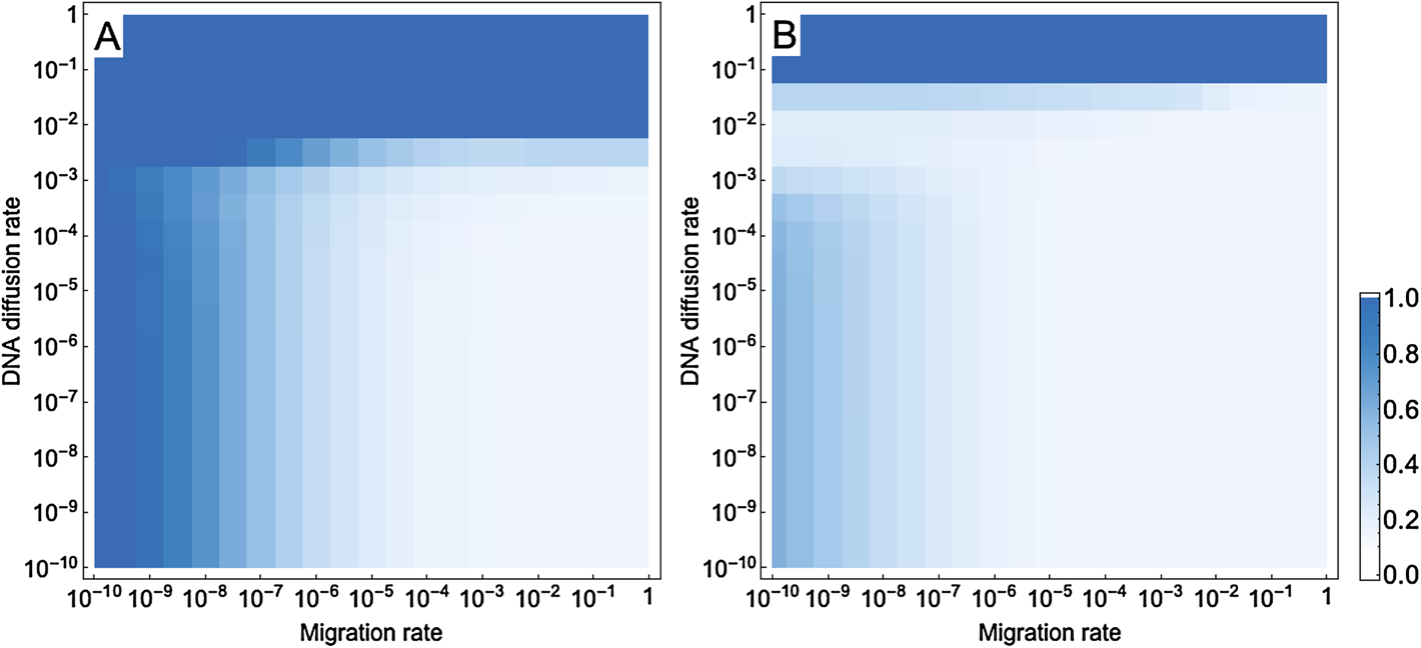
**The total frequency of bacteria of genotype 1 in transforming populations growing in a 21 × 21 space at time point 5000 with different values for bacterial migration and DNA diffusion rates and with two transformation rates: A)**  ***u*** **= 10**^**- 5**^**and B)*u*** **= 10**^**- 3**^**.** Simulations start with a bacterium of genotype 0 located at the center of the space. Each plot shows the frequencies of allele 1 for 20 parameter values of the bacterial migration rate and the DNA diffusion rate. Other parameters take the values *r* = 0.1, *K* = 100, *m* = 10^- 2^, *s* = 0.01, *μ* = 10^- 6^, *c* = 0.

In order to better understand the impact of migration on the evolutionary dynamics of our model, it may be helpful to first consider the two extreme ends of the spectrum of migration rates. In the complete absence of bacterial migration, there is only growth and adaptation in the initial grid cell, and this single subpopulation behaves as a planktonic population. Conversely, when migration rates are extremely high, the entire population becomes well mixed and allele frequencies will be the same in all grid cells. Both of these extreme cases can thus be understood from previous studies examining the impact of natural transformation in planktonic populations where the “bad genes” effect was previously described [15,21].To understand how population structure affects the “bad genes” effect, we measured the overall frequency of bacteria of genotype 1 across the whole population at one specific time point, for different intermediate migration rates as well as different DNA diffusion rates and transformation rates (Figure 3). As anticipated, the bad genes effect becomes more pronounced with increasing transformation rate. With decreasing bacterial migration rate, the “bad genes” effect is mitigated and adaptation proceeds faster. This is because the lower the migration rate, the longer it takes until new grid cells are colonized. This delayed colonization of new grid cells means that migrants spreading in new grid cells will be better adapted to start with and also encounter fewer deleterious alleles in the free DNA pool. With low migration rates adaptation is therefore impeded by the “bad genes” effect in grid cells close to the initial one but proceeds at an increasingly faster pace at the frontier of the spatially expanding population. Only at extremely low migration rates (results not shown in Figure 3) is adaptation again impeded because the bacteria fail to colonize any grid cell other than the initial one over the time period under consideration.DNA diffusion potentially has two opposing effects on the effect of transformation on adaptation. On the one hand, DNA diffusion spreads the free DNA of type 0 in the population, and thus, impedes adaptation. On the other hand, DNA diffusion depletes free DNA of type 0 and thus mitigates the bad genes effect. These opposing effects can result in a non-monotonic impact of DNA diffusion on the transformation impact, particularly with low bacterial migration rates (Figure 3B). High levels of migration make the non-monotonic effect of DNA diffusion disappear since in this case, the bacterial population behaves like a planktonic population in which the bad genes effect is pronounced. At very high DNA diffusion rates, the free DNA becomes unavailable to the bacteria, and thus the bad genes effect disappears.

In the Appendix, we present the results of the simulations in the presence of different levels of DNA degradation. Our results demonstrate that DNA degradation monotonically reduces the bad genes effect but a relatively high DNA decay rate (greater than 1/100 of the bacteria growth rate) is required to completely remove the effect of transformation (Additional file 1: Figure S1). Thus, the dynamics of transformation mentioned above are conceivable to be observed in natural circumstances.

### Adaptation with density-dependent selection

**Figure 4:**
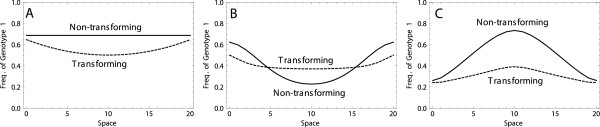
**The frequency of bacteria of genotype 1 across the equator section of a 21 × 21 grid, for non-transforming (solid curves) and transforming (dashed curves) populations growing under different density dependent selection regimes: A)** ***s***_***e***_ **= 0.01,*s***_***f***_ **= 0.01, B)*s***_***e***_ **= 0.01,*s***_***f***_ **= 0and C)*s***_***e***_ **= - 0.01,*s***_***f***_ **= 0.01.** The curves show the frequencies of allele 1 at time points: A) *T* = 1000, B) *T* = 1250 and C) *T* = 5000. (As the speed of adaptation is different in the different selection regimes, different time points have been used to informatively represent the dynamics in each regime.) Simulations start from a bacterium of type 0 at the center of the space. Interpolation was used to obtain a continuous distribution curve from discrete data points. Other parameters take the values  *r* = 0.1, *K* = 100, *m* = 10^- 2^, *u* = 10^- 3^, *μ* = 10^- 6^, *g* = 10^- 1^, *c* = 0.

In spatially structured bacterial populations, selection is often expected to be heterogeneous across bacteria occupying different locations, depending on the density of the surrounding subpopulations. For example, restriction on the diffusion of an antibiotic makes the interior bacteria less susceptible to the antibiotic, thus decreasing the strength of selection for resistance genes. In an extreme case where no antibiotics reach the interior, there might even be selection against resistance genes due to costs of resistance, i.e., not only the strength but also the direction of selection might depend on the density of the surrounding subpopulations. Similar effects may arise if diffusion of nutrients is restricted by the bacteria.To emulate such effects, we next consider the dynamics of adapting populations under selection regimes that are dependent on population density in the vicinity of a focal grid cell. We consider the effect of transformation on the distribution of genotype 1 in space in three scenarios. The first scenario is the density-independent regime where genotype 0 is always disfavored by selection. As anticipated in this case, transformation reduces the frequency of genotype 1 especially in the center of the transforming population during adaptation, due to the bad genes effect (Figure 4A).The second scenario describes a density dependent regime where genotype 0 is selected against in an empty space but becomes selectively neutral in a full space. In this case, genotype 1 becomes more frequent at the less dense frontier parts of the population in the absence of transformation. DNA diffusion mixes free DNA fragments of type 0 and type 1 that are over-represented in the interior and frontier parts of the population, respectively. Transformation then reduces the number of bacteria of genotype 1 in the frontier of the population but increases them in the internal part (Figure 4B).The third scenario features a density dependent regime where genotype 0 is favored in a free space but selected against as population density increases. This results in an overrepresentation of bacteria of genotype 1 in the interior of the population (Figure 4C). Since genotype 0 becomes more disfavored as population density increases, the free DNA of type 0 becomes frequent in the environment. Transformation with free DNA of type 0 lowers the frequency of genotype 1 in the population (Figure 4C). Overall, the results of this section indicate that in addition to a constant selection regime, transformation also affects adaptation when the population is subject to heterogeneous selective forces.

### Competition between two species

**Figure 5:**
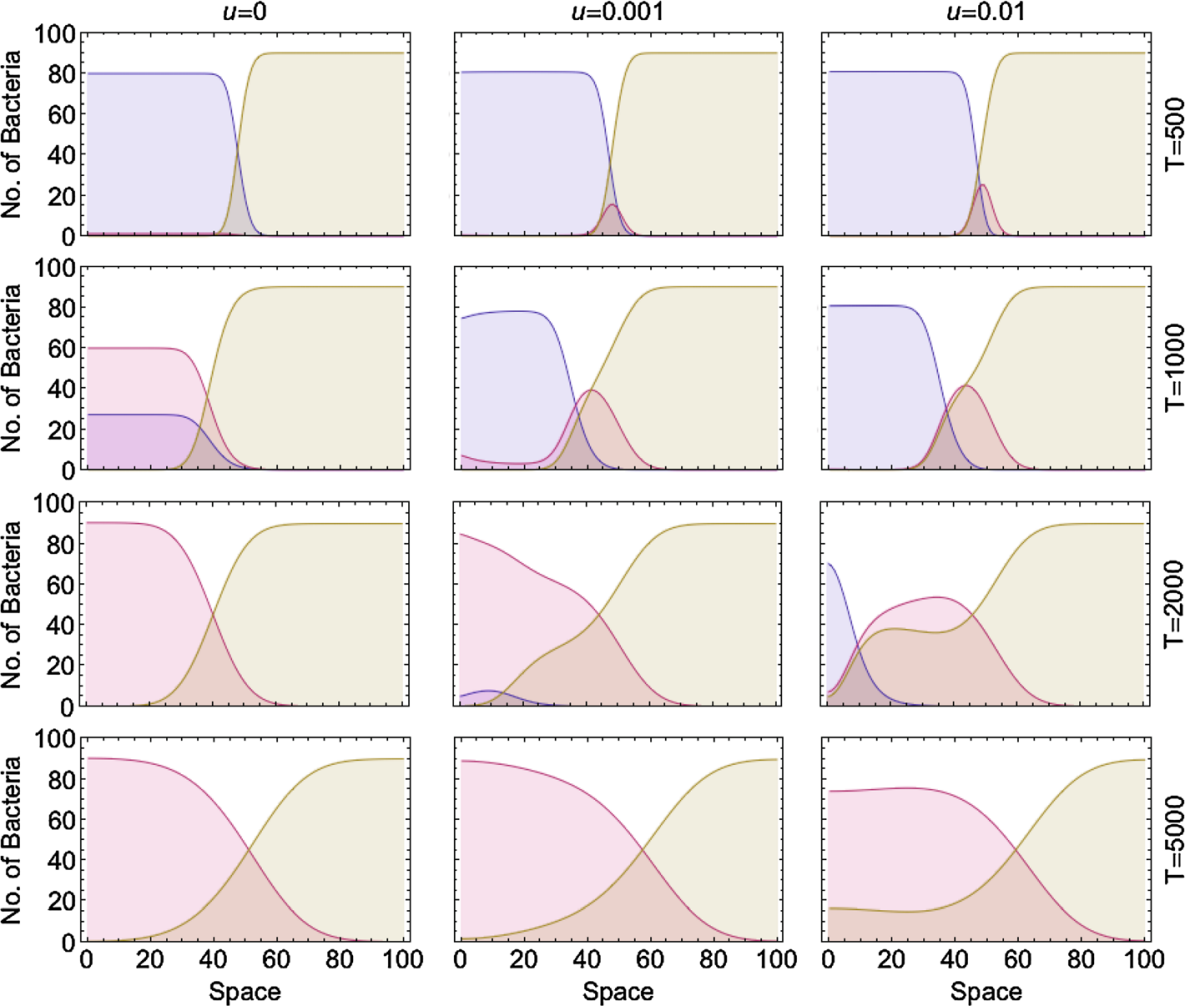
**The number of bacteria along the equator layer of a 5 × 100 space in two adapting and adapted bacterial populations at three transformation rates for the adapting type: *u*** **= 0,*u*** **= 10**^**- 3**^**and*u*** **= 10**^**- 2**^**.** The non-transforming population (brown) has already adapted to the environment and grows from a bacterium of genotype 1 located at position (3,80). The adapting population consists of two genotypes (0: blue, 1: red) and grows from a bacterium located at position (3,20). The frequencies of different genotypes are taken at time points 500, 1000, 2000 and 5000. Parameters take value: *r* = 0.1  for the adapted population and *r* = 0.105  for the adapting population, *K* = 100, *m* = 10^- 2^, *s* = 0.01, *v* = 10^- 2^, *μ* = 10^- 6^, *g* = 10^- 1^, *c* = 0.

Thus far, we have assumed that one bacterial species colonizes an empty environment, and therefore, free DNA originates from a single colony. However, in natural situations bacteria may face already adapted competitor species, so that transforming bacteria are exposed to the exogenous DNA fragments originating from the adapted species. To examine how transformation affects adaptation in this case, we study the competition between a non-transforming adapted population and an adapting population, which potentially undergoes transformation. The two populations grow across space under a constant selection regime. The non-transforming population carries allele 1 and is thus assumed to be well adapted to the environment; no mutations occur in this population. The adapted population therefore supplies the DNA fragment pool with only the beneficial allele 1. The adapting population consists of two genotypes 0 and 1 and undergoes mutation and potentially transformation. This population has a higher growth rate than the non-transforming population. However, bacteria of genotype 0 in the adapting population are selected against and are disfavored in competition with the adapted non-transforming bacteria.The two populations grow from different parts of the grid and compete with each other. Figure 5 reveals that in the absence of transformation, the adapted species is initially favored and occupies most of the space. This happens because of selection against genotype 0 in the adapting population. As adaptation proceeds, the adapting population outcompetes the non-transforming population, owing to its higher growth rate.Transformation has two opposing effects on adaptation in the adapting population: transformation with the free DNA of type 0 released by the adapting species impedes adaptation (the bad genes effect), but transformation with the free DNA of type 1, which is released by the adapted species and diffuses in space, facilitates adaptation. These opposing effects result in varying spatiogenetic patterns in different parts of the transforming population over time (Figure 5). The bacteria at the frontier of the transforming population are the first to become exposed to the beneficial free DNA of type 1, and therefore, undergo adaptation faster than the internal bacteria, which are predominantly exposed to the conspecific DNA of type 0. Adaptation continues in the transforming population and more bacteria of genotype 1 are formed. In the long term, owing to their higher growth rate, the transforming population gradually out-competes the non-transforming type.With a high compared to a low transformation rate, although transformation generates more bacteria of genotype 1 in the frontline of the population, the bad genes effect also becomes more pronounced and this outweighs the benefit of transformation. As a result, adaptation is considerably decelerated in the transforming population (Figure 5). Altogether we conclude that transformation allows bacteria to take advantage of the beneficial free DNA, especially at the frontier of the population, and this can outweigh the bad genes effect.

## Discussion

Lysed bacteria are known to supply the free DNA pool in the environment [7,23-25]. This is thought to give rise to the “bad genes effect” of natural transformation, according to which there is an overrepresentation of deleterious mutations released by selectively dead cells into the DNA pool. Previous models of planktonic bacteria considering the dynamics of a free DNA pool have shown that this effect results in a fitness cost of natural transformation that often overcomes potential benefits of transformation [16,21]. Our model shows that spatial structure of bacterial populations in combination with DNA diffusion strongly affects the bad genes effect. Specifically, we found that the bad genes effect is mostly mitigated for intermediate bacterial migration and either very high or low DNA diffusion rates.

Our model rests on the assumption that the genetic composition of the DNA fragment pool can be different from that of the cell population. This is justified because free DNA is known to persist in the environment for long periods of time and can thus to some extent retain a record of genes from previous generations [23]. In a spatial context, this also means that DNA diffusion has the potential to disseminate genes within the population, thereby enabling gene flow without actual bacterial migration. However, DNA diffusion may be restricted to varying degrees. For instance, extracellular DNA is known to serve as an abundant structural polymer in biofilms and to be required for the integrity and stability of the extracellular matrix during biofilm formation [12,13]. Besides, bacteria can utilize free DNA as a potential nutrient [20,26]. These and other factors can potentially limit the diffusion of free DNA in the environment, localize the effect of transformation and consequently reduce the extent to which transformation increases the level of genotypic homogeneity across the population.

Beyond a transient effect of slowing down adaptation, transformation may also lead to a persistent reduction in mean fitness of the population when there is spatial heterogeneity, caused by the biofilm structure, in selection pressure. One possible scenario that we examined assumes that selection for an allele becomes weaker or even negative at the center of a growing population. A case in point is the situation seen in biofilms where bacteria in the interior are often immune to the action of antibiotics (due to low accessibility of antibiotics [27] or reduced metabolism caused by nutrients depletion [28,29]), so that resistance genes are only selected for at the exterior of the biofilm. Such heterogeneous selection can entail a migration load, i.e. a reduction in the mean fitness of the population due to migration of non-adapted individuals. Our results show that transformation can produce an additional fitness load incurred through incorporating DNA that has diffused from parts of the population that are under a different selection pressure.

We also explored a situation where transformation can become beneficial during adaptation due to the acquisition of free DNA fragments originating from other, already adapted populations in the environment. For example, consider a situation where a pathogen invades and adapts to a complex environment like the nasopharynx where the pathogen needs to compete with other bacteria and is exposed to environmental stresses, such as drug treatment. Our results suggest that in a multispecies environment transformation becomes advantageous by allowing bacteria to take advantage of the exogenous DNA in the shared DNA pool that originated from already adapted species. In line with this, prior studies have shown that the rate of transformation increases during colonization of the nasopharynx by multiple strains of pneumococci [30,31]. This effect of free DNA originating from already adapted populations resembles the proposed benefit of transformation in using beneficial genes that the DNA pool retains from the past (‘the genetic time travel effect’) [32]. Similarly, Szollosi et al. [33] suggested that horizontal gene transfer through bacterial migration may be beneficial by enabling bacteria to acquire genes from other spatially distinct populations to readapt faster to changing conditions.

Our model does not include evolution at multiple loci, and therefore, does not involve any potential benefit of transformation due to the break up of non-random associations between alleles. Recombination can be beneficial during adaptation by bringing together beneficial mutations that are likely to arise on different backgrounds in finite populations (the Fisher-Muller effect) [34,35]. Martens and Hallatschek [36] showed that recombination accelerates adaptation in spatially structured populations through the Fisher-Muller effect, but their model did not incorporate a free DNA pool. As an additional feature of bacterial recombination, our results suggest that transformation with the free DNA pool potentially affects the fixation of beneficial alleles in structured populations. These findings highlight the importance of considering the dynamics of the free DNA pool to fully account for the dynamics of recombination in bacteria.

It should be noted that in our simulations we neglected the possibility that genes involved in DNA uptake and recombination themselves are subject to homologous recombination. In particular, transformation with non-functional alleles of such genes released into the environment by the non-competent bacteria will produce a self-reinforcing drive against transformation so that in the absence of any benefits of transformation the competent genotype will ultimately become extinct [37].

Although our model aims to provide insights into the dynamics of spatially structured bacterial populations undergoing transformation, our model is not specifically tailored to describe bacterial biofilms. Several previous models provide a much more realistic description of biofilm growth. For example, these models explicitly track the concentration of different nutrients and growth limiting/stimulating compounds in different layers of the biofilm and account for heterogeneity in position, biophysical and physiological properties of bacteria during biofilm formation over time see [38] for a review. Incorporating evolutionary dynamics such as those involving natural transformation into such individual-based biofilm models represents a substantial conceptual and computational challenge for future studies.

## Conclusions

This study was an attempt to elucidate the connection between horizontal gene transfer and formation of structured population in bacteria. There is growing interest among molecular microbiologists to understand this connection as researchers have realized that most bacteria live in highly structured populations and horizontal gene transfer plays a major role in determining the biological repertoire of microorganisms. Experimental tests of the above models are needed to gain a better understanding of the evolutionary dynamics of bacteria during colonization of new environments.

## Methods

In order to model evolution in a spatially structured population of bacteria, we consider a two-dimensional *L* × *P* grid of cells. In each of these grid cells, the bacteria grow at a growth rate *r* that is restricted by the same carrying capacity *K*. The bacteria migrate to adjacent grid cells at a rate *v*, and we consider all bacteria in the 8-cell neighborhood as adjacent. There is no migration beyond the borders of the grid.

We assume each bacterium has a single locus with two alleles (0 and 1) and the bacteria undergo forward (1 → 0) and backward (0 → 1) mutations at the same rate *μ*. The bacteria die at a certain rate that depends on their allelic state (see below). Upon death, each bacterium releases its genomic allele into the medium (initially the same subpopulation). In the main text, we assume that DNA persists in the environment (no degradation) but may diffuse between grid cells at a rate *g*. In contrast to bacterial migration, we assume that the DNA may diffuse out of the grid of subpopulations.

The bacteria undergo natural transformation at a rate *u* per bacterium and encounter DNA molecules present in the same grid cell. An imported DNA fragment replaces the genomic allele through homologous recombination. This may lead to the conversion of a genotype to the other type (provided the allele that is taken up is different from the original one), and it also leads to a reduction of fragments in the pool of free DNA. Besides DNA diffusion and transformation, other environmental factors can limit the availability of environmental DNA, such as DNA degradation and DNA consumption by bacteria as a nutrient (see Discussion). To account for this, we include a rate - *c* - at which DNA becomes unavailable to the bacteria. The effect of this parameter is studied in the Appendix.

Taken together, the dynamics of the bacterial population and the DNA pool are described by the following system of differential equations:

(1) $$\begin{array}{l} {\dot{x}}_{\mathrm{ij}}^{0}=rx_{\mathrm{ij}}^{0}\left(1-\frac{x_{\mathrm{ij}}^{0}+x_{\mathrm{ij}}^{1}}{K}\right)-\left(m+s_{\mathrm{ij}}\right)x_{\mathrm{ij}}^{0}+v\left(\sum_{p,q\;\in \;N\left(i,j\right)}x_{\mathrm{pq}}^{0}-n\left(i,j\right)x_{\mathrm{ij}}^{0}\right)\\ \;+\mu \left(x_{\mathrm{ij}}^{1}-x_{\mathrm{ij}}^{0}\right)+u(d_{\mathrm{ij}}^{0}x_{\mathrm{ij}}^{1}-d_{\mathrm{ij}}^{1}x_{\mathrm{ij}}^{0})\\ {\dot{x}}_{\mathrm{ij}}^{1}=rx_{\mathrm{ij}}^{1}\left(1-\frac{x_{\mathrm{ij}}^{0}+x_{\mathrm{ij}}^{1}}{K}\right)-mx_{\mathrm{ij}}^{1}+v\left(\sum_{p,q\;\in \;N\left(i,j\right)}x_{\mathrm{pq}}^{1}-n\left(i,j\right)x_{\mathrm{ij}}^{1}\right)+\mu \left(x_{\mathrm{ij}}^{0}-x_{\mathrm{ij}}^{1}\right)\\ \;+\;u\left(d_{\mathrm{ij}}^{1}x_{\mathrm{ij}}^{0}-d_{\mathrm{ij}}^{0}x_{\mathrm{ij}}^{1}\right)\\ {\dot{d}}_{\mathrm{ij}}^{0}=\left(m+s_{\mathrm{ij}}\right)x_{\mathrm{ij}}^{0}-ud_{\mathrm{ij}}^{0}\left(x_{\mathrm{ij}}^{0}+x_{\mathrm{ij}}^{1}\right)+g(\sum_{p,q\;\in \;N\left(i,j\right)}d_{\mathrm{pq}}^{0}-8d_{\mathrm{ij}}^{0})-cd_{\mathrm{ij}}^{0}\\ {\dot{d}}_{\mathrm{ij}}^{1}=mx_{\mathrm{ij}}^{1}-ud_{\mathrm{ij}}^{1}\left(x_{\mathrm{ij}}^{0}+x_{\mathrm{ij}}^{1}\right)+g(\sum_{p,q\;\in \;N\left(i,j\right)}d_{\mathrm{pq}}^{1}-8d_{\mathrm{ij}}^{1})-cd_{\mathrm{ij}}^{1} \end{array}$$

In these equations, *x* and *d* denote the number of bacteria and DNA fragments, respectively. Subscripts indicate the grid cell and superscripts indicate the allelic state. The term *N*(*i*, *j*) denotes the set of neighboring grid cells of the grid cell with the coordinates *i* and *j* and the term *n*(*i*, *j*) represents the number of these neighboring grid cells (i.e., *n*(*i*, *j*) = |*N*(*i*, *j*)|).

Natural selection operates through differences in death rates between the two bacterial genotypes. The total death rate is composed of an intrinsic death rate *m* that is the same for all bacteria and a selection coefficient that can depend on both genotype and population density. Specifically, we consider two different selection regimes. In the first regime, the allele 0 entails a death rate that is increased by a constant amount *s*. Although genotype 0 may still grow and establish a population, selection thus reduces the net growth rate of genotype 0 (*r-m-s*) by an amount *s* compared to the favoured genotype 1. Moreover, selection also decreases the equilibrium population size of genotype 0 (*K*(*r*-*m*-*s*)/*r vs. K*(*r-m*)/*r*).

In the second selection regime, we assume that this difference in death rate depends on the population density in the surrounding grid cells. For bacteria located in a completely empty neighborhood of *l* adjacent grid cells, allele 0 entails a death rate that is changed by an amount *s*_*e*_ (which can also take negative values). With increasing bacterial density in this neighborhood, this selection coefficient changes linearly until it reaches a value of *s*_*f*_ if the neighborhood is completely filled with bacteria. Hence, increase in death rate of genotype 0 is described by:

(2) $$\begin{array}{l} s_{\mathrm{ij}}=s_{e}\left(1-\frac{1}{\left|H_{l}\left(i,j\right)\right|}\sum_{p,q\in \;H_{l}\left(i,j\right)}\frac{x_{\mathrm{pq}}^{0}+x_{\mathrm{pq}}^{1}}{K}\right)\\ \;+s_{f}\left(\frac{1}{\left|H_{l}\left(i,j\right)\right|}\sum_{p,q\in \;H_{l}\left(i,j\right)}\frac{x_{\mathrm{pq}}^{0}+x_{\mathrm{pq}}^{1}}{K}\right) \end{array}$$

Here *H*_*l*_(*i*, *j*) is the set of *l* grid cells forming a neighborhood of grid cell (*i*, *j*); this also includes the grid cell (*i*, *j*) itself. The term |*H*_*l*_(*i*, *j*)| denotes the size of *H*_*l*_(*i*, *j*). The parameters used in the model and their units are listed in Table 1.

**Table 1:** Parameters used in the model

**Parameter**	**Description**	**Unit**
*L* and *P*	Numbers of grid cells in two dimensional space	–
*K*	Carrying capacity of each grid cell	–
*r*	Growth rate	Per bacterium per unit time
*μ*	Mutation rate	Per bacterium per unit time
*m*	Intrinsic death rate	Per bacterium per unit time
*u*	Transformation rate	Per bacterium per DNA fragment per unit time
*v*	Bacterial migration rate	Per bacterium per unit time
*g*	DNA diffusion rate	Per DNA fragment per unit time
*s*	Selection coefficient	Per bacterium per unit time
*c*	DNA decay rate	Per DNA fragment per unit time

We chose a realistic range of parameters for the parameters that can be approximated experimentally, like the mutation and recombination rate. For other parameters, such as the rates of DNA diffusion and bacterial migration in a biofilm for which there are no exact experimental estimates, the parameter space was screened. We assumed a mutation rate of 10^-6^. For the transformation rate, there is wide variation in experimentally approximated transformation frequencies (10^-6^-10^-3^ for *H. pylori*[39] and 10^-6^-10^-2^ for *Streptococcus pneumonia*[40]). Here we used transformation rate of 10^-3^-10^-5^ (per bacterium per DNA fragment per unit time) in most simulations. We also studied the effect of higher transformation rates in some simulations.

We examined the impact of transformation on adaptation in the course of colonization of a new environment. The initial bacterium had the genotype “0”. We then followed the eco-evolutionary dynamics by numerically solving the above system of equations using the software package Mathematica v.8 (Wolfram Research Inc. [41]).

## Competing interests

The authors declare that they have no competing interests.

## Authors’ contribution

DM and JE conceived the research, analyzed the results and wrote the paper. DM performed the research. Both authors read and approved the final manuscript.

## Supplementary Material

Additional file 1: Figure S1 The effect of DNA decay on adaptation rate. Click here for file

## References

[B1] Costerton W Veeh R Shirtliff M Pasmore M Post C Ehrlich G The application of biofilm science to the study and control of chronic bacterial infections J Clin Invest 2003 112 10 1466 1477 10.1172/JCI200320365 14617746 PMC259139

[B2] Hall-Stoodley L Costerton JW Stoodley P Bacterial biofilms: from the natural environment to infectious diseases Nat Rev Microbiol 2004 2 2 95 108 10.1038/nrmicro821 15040259

[B3] Rendueles O Ghigo JM Multi-species biofilms: how to avoid unfriendly neighbors FEMS Microbiol Rev 2012 36 5 972 989 22273363 10.1111/j.1574-6976.2012.00328.x

[B4] Fux CA Costerton JW Stewart PS Stoodley P Survival strategies of infectious biofilms Trends Microbiol 2005 13 1 34 40 10.1016/j.tim.2004.11.010 15639630

[B5] Vos M Why do bacteria engage in homologous recombination? Trends Microbiol 2009 17 6 226 232 10.1016/j.tim.2009.03.001 19464181

[B6] Madsen JS Burmolle M Hansen LH Sorensen SJ The interconnection between biofilm formation and horizontal gene transfer Fems Immunol Med Mic 2012 65 2 183 195 10.1111/j.1574-695X.2012.00960.x 22444301

[B7] Marks LR Reddinger RM Hakansson AP High levels of genetic recombination during nasopharyngeal carriage and biofilm formation in streptococcus pneumoniae Mbio 2012 3 5 e00200 12 23015736 10.1128/mBio.00200-12 PMC3448161

[B8] Grande R Di Campli E Di Bartolomeo S Verginelli F Di Giulio M Baffoni M Bessa LJ Cellini L Helicobacter pylori biofilm: a protective environment for bacterial recombination J Appl Microbiol 2012 113 3 669 676 10.1111/j.1365-2672.2012.05351.x 22639839

[B9] Molin S Tolker-Nielsen T Gene transfer occurs with enhanced efficiency in biofilms and induces enhanced stabilisation of the biofilm structure Curr Opin Biotechnol 2003 14 3 255 261 10.1016/S0958-1669(03)00036-3 12849777

[B10] Li YH Lau PC Lee JH Ellen RP Cvitkovitch DG Natural genetic transformation of Streptococcus mutans growing in biofilms J Bacteriol 2001 183 3 897 908 10.1128/JB.183.3.897-908.2001 11208787 PMC94956

[B11] Petersen FC Tao L Scheie AA DNA binding-uptake system: a link between cell-to-cell communication and biofilm formation J Bacteriol 2005 187 13 4392 4400 10.1128/JB.187.13.4392-4400.2005 15968048 PMC1151753

[B12] Matsukawa M Greenberg EP Putative exopolysaccharide synthesis genes influence Pseudomonas aeruginosa biofilm development J Bacteriol 2004 186 14 4449 4456 10.1128/JB.186.14.4449-4456.2004 15231776 PMC438629

[B13] Whitchurch CB Tolker-Nielsen T Ragas PC Mattick JS Extracellular DNA required for bacterial biofilm formation Science 2002 295 5559 1487 10.1126/science.295.5559.1487 11859186

[B14] Cohen E Kessler DA Levine H Recombination dramatically speeds up evolution of finite populations Phys Rev Lett 2005 94 9 098102 15784005 10.1103/PhysRevLett.94.098102

[B15] Moradigaravand D Engelstädter J The evolution of natural competence: disentangling costs and benefits of sex in bacteria Am Nat 2013 182 4 E112 126 10.1086/671909 24021408

[B16] Moradigaravand D Engelstädter J The effect of bacterial recombination on adaptation on fitness landscapes with limited peak accessibility Plos Comput Biol 2012 8 10 e1002735 10.1371/journal.pcbi.1002735 23133344 PMC3487459

[B17] Levin BR Cornejo OE The population and evolutionary dynamics of homologous gene recombination in bacteria Plos Genet 2009 5 8 e1000601 10.1371/journal.pgen.1000601 19680442 PMC2717328

[B18] Wylie CS Trout AD Kessler DA Levine H Optimal strategy for competence differentiation in bacteria Plos Genet 2010 6 9 e1001108 10.1371/journal.pgen.1001108 20838595 PMC2936531

[B19] Michod RE Bernstein H Nedelcu AM Adaptive value of sex in microbial pathogens Infect Genet Evol 2008 8 3 267 285 10.1016/j.meegid.2008.01.002 18295550

[B20] Redfield RJ Do bacteria have sex? Nat Rev Genet 2001 2 8 634 639 10.1038/35084593 11483988

[B21] Redfield RJ Evolution of bacterial transformation - is sex with dead cells ever better than no sex at all Genetics 1988 119 1 213 221 3396864 10.1093/genetics/119.1.213 PMC1203342

[B22] Redfield RJ Schrag MR Dean AM The evolution of bacterial transformation: sex with poor relations Genetics 1997 146 1 27 38 9135998 10.1093/genetics/146.1.27 PMC1207942

[B23] Nielsen KM Johnsen PJ Bensasson D Daffonchio D Release and persistence of extracellular DNA in the environment Environ Biosafety Res 2007 6 1–2 37 53 17961479 10.1051/ebr:2007031

[B24] Lewis K Programmed death in bacteria Microbiology and molecular biology reviews : MMBR 2000 64 3 503 514 10.1128/MMBR.64.3.503-514.2000 10974124 PMC99002

[B25] Mortier-Barriere I de Saizieu A Claverys JP Martin B Competence-specific induction of recA is required for full recombination proficiency during transformation in Streptococcus pneumoniae Mol Microbiol 1998 27 1 159 170 10.1046/j.1365-2958.1998.00668.x 9466264

[B26] Finkel SE Kolter R DNA as a nutrient novel role for bacterial competence gene homologs J Bacteriol 2001 183 21 6288 6293 10.1128/JB.183.21.6288-6293.2001 11591672 PMC100116

[B27] Anderl JN Franklin MJ Stewart PS Role of antibiotic penetration limitation in Klebsiella pneumoniae biofilm resistance to ampicillin and ciprofloxacin Antimicrob Agents Chemother 2000 44 7 1818 1824 10.1128/AAC.44.7.1818-1824.2000 10858336 PMC89967

[B28] Anderl JN Zahller J Roe F Stewart PS Role of nutrient limitation and stationary-phase existence in Klebsiella pneumoniae biofilm resistance to ampicillin and ciprofloxacin Antimicrob Agents Chemother 2003 47 4 1251 1256 10.1128/AAC.47.4.1251-1256.2003 12654654 PMC152508

[B29] Gilbert P Collier PJ Brown MR Influence of growth rate on susceptibility to antimicrobial agents: biofilms, cell cycle, dormancy, and stringent response Antimicrob Agents Chemother 1990 34 10 1865 1868 10.1128/AAC.34.10.1865 2291653 PMC171955

[B30] Donkor ES Bishop CJ Antonio M Wren B Hanage WP High levels of recombination among Streptococcus pneumoniae Isolates from the Gambia Mbio 2011 2 3 e00040 11 21693638 10.1128/mBio.00040-11 PMC3119534

[B31] Leung MHY Oriyo NM Gillespie SH Charalambous BM The adaptive potential during nasopharyngeal colonisation of Streptococcus pneumoniae Infect Genet Evol 2011 11 8 1989 1995 10.1016/j.meegid.2011.09.002 21925618

[B32] Engelstädter J Moradigaravand D Adaptation through genetic time travel? Fluctuating selection can drive the evolution of bacterial transformation Proc R Soc Lond B 2014 281 1775 20132609 10.1098/rspb.2013.2609 PMC3866407 24285199

[B33] Szollosi GJ Derenyi I Vellai T The maintenance of sex in bacteria is ensured by its potential to reload genes Genetics 2006 174 4 2173 2180 10.1534/genetics.106.063412 17028325 PMC1698621

[B34] Muller HJ Some genetic aspects of sex Am Nat 1932 66 118 138 10.1086/280418

[B35] Fisher RA The Genetical Theory Of Natural Selection 1930 USA: Oxford University Press

[B36] Erik A Interfering waves of adaptation promote spatial mixing Martens and Oskar Hallatschek Genet 2011 189 3 1045 1060 doi: 10.1534/genetics.111.130112. http://www.ncbi.nlm.nih.gov/pmc/articles/PMC3213383/10.1534/genetics.111.130112 PMC3213383 21900264

[B37] Redfield RJ Genes for breakfast: the have-your-cake-and-eat-it-too of bacterial transformation J Hered 1993 84 5 400 404 8409360 10.1093/oxfordjournals.jhered.a111361

[B38] Kreft JU Picioreanu C Wimpenny JWT van Loosdrecht MCM Individual-based modelling of biofilms Microbiol-Sgm 2001 147 2897 2912 10.1099/00221287-147-11-2897 11700341

[B39] Wang Y Roos KP Taylor DE Transformation of Helicobacter pylori by chromosomal metronidazole resistance and by a plasmid with a selectable chloramphenicol resistance marker J Gen Microbiol 1993 139 10 2485 2493 10.1099/00221287-139-10-2485 8254319

[B40] Evans BA Rozen DE Significant variation in transformation frequency in Streptococcus pneumoniae Isme J 2013 7 4 791 799 10.1038/ismej.2012.170 23303370 PMC3603394

[B41] Wolfram Research, Inc Mathematica, Version 8.0 2010 Champaign, IL

