# Supplementary material for: The impact of natural transformation on adaptation in spatially structured bacterial populations
Source: BMC Evol Biol. 2014 Jun 20;14:141. doi: 10.1186/1471-2148-14-141 (PMC4080760; doi:10.1186/1471-2148-14-141)
Supplement: Additional file 1: Figure S1 — The effect of DNA decay on adaptation rate. [file 1471-2148-14-141-S1.pdf]

The impact of natural transformation on adaptation in spatially structured bacterial populations

Danesh Moradigaravand & Jan Engelstädter

Effect of DNA decay on adaptation rate

In this part we study the effect of DNA decay on the rate of adaptation in spatially restricted populations. To this end we repeated the simulations in Figure 3 with different levels of DNA degradation and subsequently measured the total frequency of bacteria with allele 1 at one time point to estimate the rate of adaptation. As anticipated, DNA decay weakens the bad genes effect in a monotonic way as the free DNA becomes unavailable to the bacteria. The bad genes effect dissipates at higher DNA decay rates with increasing transformation rate because here the free DNA is taken up more quickly and thus persists for shorter time periods in the environment. Our results also reveal that the bad genes effect is still pronounced if DNA decay rate is as high as 1/100 of the bacterial growth rate (Figure S1C and S1F). DNA stability varies greatly in different environments but ranges from several hours to weeks in soil and water [1-5]. Therefore environmental DNA is relatively persistent in bacterial environments compared to bacterial generation times and can influence adaptation on the time scales studied here.

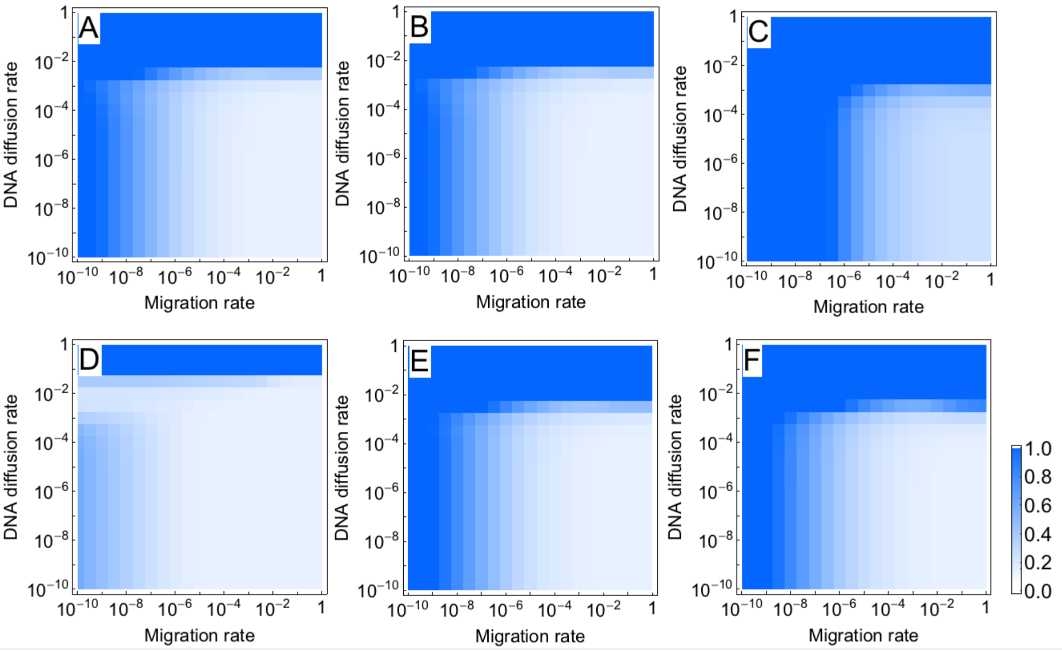

Figure S1. The total frequency of bacteria of genotype 1 in transforming populations growing in a 21x21 space at time point 5000 with different values for bacterial migration and DNA diffusion rates at three DNA decay rates: (A, D) $c = 0$, (B, E) $c = 0.005$ and (C, F) $c = 0.001$. Figures (A, B, C) and (D, E, F) correspond to transformation rates $u = 0.00001$ and $u = 0.001$, respectively. Simulations start with a bacterium of genotype 0 located at the center of the space. Each plot shows the frequencies of allele 1 for 20 parameter values of the bacterial migration rate and the DNA diffusion rate. Other parameters take the values $r = 0.1, K = 100, m = 10^{-2}, s = 0.01, \mu = 10^{-6}$.

The impact of natural transformation on adaptation in spatially structured bacterial populations

Danesh Moradigaravand & Jan Engelstädter

1. Alvarez AJ, Yumet GM, Santiago CL, Toranzos GA: **Stability of manipulated plasmid DNA in aquatic environments**. *Environmental Toxicology and Water Quality* 1996, **11**(2):129-135.
2. Levy-Booth DJ, Campbell RG, Gulden RH, Hart MM, Powell JR, Klironomos JN, Pauls KP, Swanton CJ, Trevors JT, Dunfield KE: **Cycling of extracellular DNA in the soil environment**. *Soil Biology & Biochemistry* 2007, **39**(12):2977-2991.
3. Dejean T, Valentini A, Duparc A, Pellier-Cuit S, Pompanon F, Taberlet P, Miaud C: **Persistence of environmental DNA in freshwater ecosystems**. *Plos One* 2011, **6**(8):e23398.
4. Romanowski G, Lorenz MG, Sayler G, Wackernagel W: **Persistence of Free Plasmid DNA in Soil Monitored by Various Methods, Including a Transformation Assay**. *Applied and environmental microbiology* 1992, **58**(9):3012-3019.
5. Nielsen KM, Johnsen PJ, Bensasson D, Daffonchio D: **Release and persistence of extracellular DNA in the environment**. *Environ Biosafety Res* 2007, **6**(1-2):37-53.
